# Effect of Hamstring Tightness and Fatigue on Dynamic Stability and Agility in Physically Active Young Men

**DOI:** 10.3390/s23031633

**Published:** 2023-02-02

**Authors:** Alberto Encarnación-Martínez, Antonio García-Gallart, Pedro Pérez-Soriano, Ignacio Catalá-Vilaplana, Julia Rizo-Albero, Roberto Sanchis-Sanchis

**Affiliations:** 1 Research Group in Sports Biomechanics (GIBD), Department of Physical Education and Sports, University of Valencia, 46010 Valencia, Spain; 2 The Civil Guard, Secretary of State for Security, Ministry of the Interior, 28010 Madrid, Spain

**Keywords:** hamstring shortening, flexibility, one-leg balance, landing

## Abstract

Hamstring extensibility has been defined as a factor to diminished dynamic stability and therefore increased risk of injury. The purpose of this study was to analyse the effects of hamstring tightness and fatigue on dynamic stability and agility. Nineteen participants were divided between the normal extensibility group (NEG) (*n* = 9, 82.2° ± 12.4°) and hamstrings tightness group (HTG) (*n* = 10, 64° ± 4.9°) using the passive straight leg raise test. To analyse dynamic stability and agility, they performed the modified Star Excursion Balance Test (mSEBT) and Dynamic Postural Stability Index (DPSI), and hexagon agility test, respectively, before and after a fatigue protocol. A two-way repeated measures ANOVA was used to determine differences among conditions: NEG vs. HTG, and rested vs. fatigued. HTG showed a significantly lower reach in the anterior direction in the mSEBT in pre- and post-fatigue than NEG. Participants in the NEG showed poor stability after landing in the mediolateral direction on DPSI post-fatigue. No significant changes were found in agility related with the group nor fatigue state. Participants with hamstring extensibility reduction has no differences in dynamic stability after landing nor agility after fatigue test, but significantly affects reaching distances during one-leg balance. As a conclusion, a reduction in range of motion in HTG was observed, but no other effects were observed on performance and dynamic stability after a local fatigue protocol depending on hamstring extensibility.

## 1. Introduction

Dynamic stability is defined as the ability to maintain the body in equilibrium by keeping the centre of mass over a base and as the ability to maintain an erect posture of the body [1]. The ability to maintain stability depends on the activity of the central nervous system (CNS), which provides quick and accurate feedback to respond to postural alterations [1]. Some studies correlate the lack of postural stability with an increased injury risk of the ankle and knee [2,3], so it is important to understand the factors that contribute to the ability to maintain stability in order to introduce injury prevention strategies.

Running related injuries, located mainly in the lower limbs, have been associated with several risk factors where ankle instability, ACL deficiency, hamstring tightness and fatigue are some of them [3]. Participants in that study appear to use compensatory strategies by changing their biomechanics while doing exercise. For example, participants with functional ankle instability tend to land in a more dorsiflexed position and use hip strategies to land from a jump [4]. This biomechanical alteration leads to muscular compensations and injuries in the long term.

Hamstring musculature has one of the highest incidences of injuries in sports that require rapid accelerations and changes of direction [5]. Mostly composed of type II fibres [5], this biarticular muscle is responsible for knee flexion and hip extension, and this double action makes it more vulnerable to strain. Limited extensibility of the hamstrings results in changes in the dynamic range of motion used while moving [6]. Hamstring tightness is one of the factors that affect dynamic balance because it loses the ability to deform while being stretched [7]. This inability results in reduced hip flexion, altered hip extension, and a posterior tilt of the pelvis, which leads to decreased lumbar lordosis and back pain [8]. Hamstring tightness also leads to increased quadriceps activity, which could lead to tendinopathies. Moorhouse and Granata [9] state that control of the lumbar spine and the lumbo-pelvic-hip complex is important to maintain stability and balance, and this is why a posteriorly tilted pelvis caused by hamstring tightness alters proprioception.

Hamstring tightness also affects performance [6] as participants with greater flexibility demonstrated more power, speed, and agility than participants with lesser hamstring flexibility. Among the variables analysed and related to sports performance, agility is one of the most common variables measured during athletic performance testing. The hexagonal agility test is commonly used by coaches to measure agility as it is a test that can be performed in a laboratory environment [10]. The differences in agility between participants with and without hamstring tightness could be related with the speed of the rate of shortening and with the muscular tendon unit stiffness [6].

Fatigue, another factor that affects dynamic balance, is also common in sports. Its presence reduces the muscle’s ability to generate force and therefore to stabilize correctly [11], probably because tightness affects the muscles’ ability to absorb and produce energy [12]. There is a high incidence of injuries at the end of competitions, and it has been related to the alterations of the neuromuscular system and the limited ability to dynamically stabilize the lower extremities [13]. Fatigue and its by-products delay afferent and efferent responses of the neuromuscular system leading to a decrease in postural control and performance [14]. It is important to know the effects that fatigue produces on the biomechanical response and how hamstring tightness can modulate that biomechanical response.

To our understanding, there is no literature studying the effects of hamstring tightness and fatigue on dynamic stability and agility, so we were interested to know if there was any relationship between hamstring tightness, the ability to stabilize in a sport-specific test like the jump and landing test and the agility of the participants. Finally, we also wanted to investigate the reach directions affected by hamstring tightness and if they change after a fatigue protocol because of the muscular compensation. Therefore, this study aimed to analyse the effects of hamstring tightness and fatigue on dynamic stability and agility. We hypothesized that: (I) dynamic stability will be reduced in participants with hamstring shortening, (II) performance in agility test will be negatively affected by hamstring tightness, and (III) fatigue will affect participants with hamstring shortening the most.

The key contributions of this paper can be summarized as follows:

- We compared the dynamic stability and agility response to a quick functional fatigue protocol to allow us to understand the response after fatigue situation.
- We also compared the interaction of hamstring tightness in the biomechanical response (dynamic stability and agility) by comparing two samples, one with hamstring tightness and another without it.
- We present here that participants with hamstring tightness have less range of motion and reduces the reaches after fatigue protocol, but no differences were found in agility performance and dynamic stability.

## 2. Materials and Methods

### 2.1. Participants

Nineteen physically active sport science students voluntarily participated in this study (Table 1) and were divided into two groups based on the existence or not of hamstring tightness following the reference criterion reported by Palmer and Epler [15] and used by Ayala et al. [16]. This criterion established a threshold in the Passive Straight-Leg Raise (PSLR) test, classifying as normal hamstring the participants who reached more or equal than 80° in that test, and tightness hamstring who reached less than 80°. The inclusion criteria were: no history of lower extremity within the last six months, no previous use of foot orthoses, and being physically active (exercise practice three sessions/week for at least 30 min/session). The final sample size was previously calculated using the G-Power 3 software (version 3.1.9.7, Düsseldorf, Germany). The results indicated that at least a sample of eight physically active sport science students was required to detect statistical differences in the variables with a minimum detectable effect size of f = 0.85 (large) (α = 0.05, β = 0.05, power = 0.97). Informed consent was provided to all participants before inclusion in the study. All the experimental procedures followed the Declaration of Helsinki principles and were approved by the University Ethics Committee (registry number: 270916).

### 2.2. Experimental Protocol

**Figure 1:**
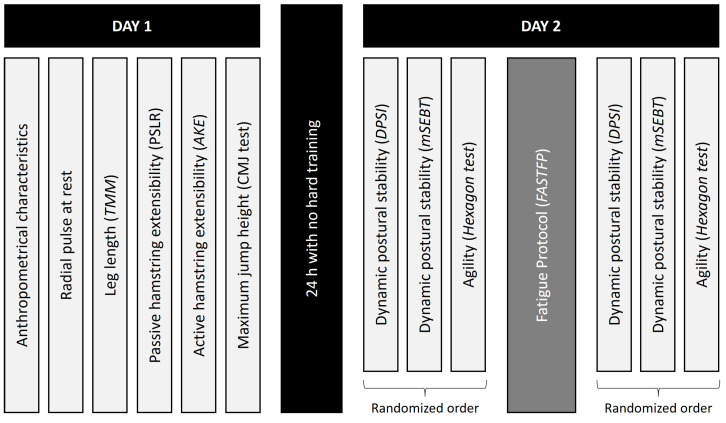
Assessment protocol used in this study.

The assessment protocol (Figure 1) was performed in two days, in the same laboratory, under similar environmental conditions (43 m altitude, 22–24 °C, 60–70% relative humidity). A 24 h period with no hard training was established between the two days. On the first day, participants’ anthropometrical characteristics, radial pulse at rest, leg length, maximum height of a jump by mean of a CMJ test, and passive and active extensibility were measured. On the second day, after a self-determined 10 min warm-up [17], dynamic stability and agility were evaluated, in a randomized order, before and after the fatigue protocol. In order to reach a fatigue condition, the Functional Agility Short-Term Fatigue Protocol (FASTFP) [18,19] was performed. So, when fatigue criteria were reached, the participant immediately performed the dynamic stability and agility tests. All the tests used to measure the above-mentioned variables are explained in depth below.

#### 2.2.1. Leg Length

Leg length was measured using the Tape Measure Method (TMM) [20], and was recorded in order to normalize the reaches in the mSEBT. An anthropometric tape (Holtain Ltd., Crymych, UK) was used to measure anthropometric dimensions. The protocol demonstrated high intertester (ICC 0.991) and intratester (ICC 0.990–0.985) reliability [19]. All participants wore clothing that allowed palpation. The leg length measurement was made with the participants in a supine position with their pelvis and leg examined in a relaxed neutral alignment. The proximal end of the tape measure was placed on the inferior aspect of the ASIS and drawn caudally across the leg toward the medial malleolus, while the distal end of the tape was placed on the inferior aspect of the medial malleolus [20,21]. In order to reduce the measurement variability, all the measures were taken by the same researcher.

#### 2.2.2. Passive Hamstring Extensibility

Passive Straight Leg Raise test (PSLR), with an intra-examiner (ICC 0.940) and intra-participant (ICC 0.946) reliability [22], was used to measure the passive hamstring muscle extensibility. The procedure used was described by Youdas et al. [21]. The participant started in a supine position lying on a mat, with the legs straight and the ankle of the tested leg in a relaxed position [23,24]. For this test, two examiners were required. The first examiner holds the unanalysed leg against the mat with one hand in order to avoid posterior pelvic rotation. With the free hand, he held the calcaneus of the tested leg and passively left the leg in a sagittal direction ensuring that the knee was straight, and the ankle was relaxed. The test stopped when the raised leg made a countermovement resistance that was perceptible to the examiner and the participant. Once this point was reached, the second examiner measured the angle formed by the tested leg and the trunk using a universal goniometer (Baseline^®^ Plastic Absolute+Axis^®^ Goniometer, 360-degree head, 12-inch arms). For this purpose, one arm of the goniometer was placed on the horizontal line of the trunk, the axis of the goniometer on the greater trochanter of the femur, and the mobile arm of the goniometer in the direction of the lateral epicondyle. Three measurements were obtained for each leg. The absolute error of the goniometer was 1°, determined by the smallest unit of measurement allowed by the system.

#### 2.2.3. Active Hamstring Extensibility

Active hamstring extensibility was measured by the Active Knee Extension test (AKE), which was performed in a hip flexion position, thus replicating the hip position during running, striking and striding. It required the participant to actively move the knee through a full range of motion so is more specific than its passive variation [25]. The protocol modified by Connor et al. [26], which has demonstrated high intertester (ICC 0.98) and intratester (ICC 0.89–0.94) reliability, was used. For the AKE, two examiners were also required. The participant was tested in a supine position on a stretcher, holding the back of the femur of the tested leg with their hands. The participant had to move their leg to a 90° hip and knee flexion position. Below the knee that was not tested, a cylindrical support was placed so that the knee forms a 20° flexion angle. In this position, the first examiner placed a vertical bar on the floor in line with the femur of the tested leg, thus forming a 90° angle to the floor. The first examiner checked that the 90° hip flexion was maintained while the second examiner placed the goniometer with the axis over the lateral epicondyle of the flexed leg, one of the arms in the direction of the femur, and the other arm in the direction of the lateral malleolus. Then, the participant had to extend the knee until a firm resistance against the movement was felt. At this point, the second examiner measured the angle formed between the femur and the tibia. This test, performed three times on each leg alternately, was considered invalid if the knee was not at 90° at the start of the contraction, the greater trochanter was not aligned with the vertical bar, the ankle was dorsiflexed, or the head was flexed.

The accuracy of the method employed to register the PSLR and AKE tests was calculated by measuring 23 angles generated by a computerized chart plotter. The average of absolute value of the differences between known and observed values was 0.13° (SD: 0.21°).

#### 2.2.4. Dynamic Postural Stability

Modified Star Excursion Balance Test (mSEBT) and Dynamic Postural Stability Index (DPSI) were used to evaluate dynamic postural stability in reach and landing tasks, respectively. According to Gribble et al. [27], mSEBT evaluates the reach in anterior, posterolateral and posteromedial directions. Intratester reliability (ICC) ranged from 0.85 to 0.89, whereas intertester reliability was nearly perfect, ranging from 0.97 to 1.00 [28]. Before the assessment, four familiarization attempts in each direction were performed [4,29]. Then, three randomized attempts in each direction were recorded [4]. The average reach in anterior, posterolateral, and posteromedial directions and the sum of the three were saved. Moreover, the reached distance was normalized to the limb length using the distance in centimetres from the anterosuperior iliac spine to the ipsilateral centre of the medial malleolus [30].

Dynamic postural stability after landing was evaluated through an adaptation of the DPSI test proposed by Wikstrom et al. [31] and Ross et al. [32], with an excellent reliability (ICC 0.91–0.98) [30]. Participants were placed 0.7 m from the centre of a force platform (Kitsler 9286BA, Kistler Group, Winterthur, Switzerland), and they were instructed to double-leg jump over an elastic band—set at 50% of their maximum jump height—with hands on hips and looking to the front, landing on their dominant limb, and stabilizing as quickly as possible. After landing, participants remained in a single-leg stance for 20 s with the first 3 s from impact being used for further analysis [33]. This impact was recognized as the vertical ground reaction forces (VGRFs) instant that exceeded 10N [33].

Fifty (50)% of their maximum jump height was calculated, 24 h before testing day, from the highest jump of three valid countermovement jumps (CMJ) [33]. For CMJ execution, participants were instructed to hold their hands on hip and to jump has hight as possible without limitations of leg flexion-extension. A minimum of three practice attempts were allowed [2], and three attempts were performed to evaluate the mediolateral, anteroposterior, vertical, and global stability indices, recording the ground reaction force signals at a frequency of 1000 Hz and using the formulas proposed by Wikstrom et al. [33]. It should be noted that these dynamic postural stability variables do not have specific units because they are dimensionless. Therefore, higher values (i.e., 0.500) indicate worse stability and lower values (i.e., 0.200) indicate better stability.

#### 2.2.5. Agility

Agility was evaluated by the Hexagon Agility test, which was adapted from Farlinger et al. [34], described in detail by Baechle and Earle [35], and used by Miranda et al. [10]. A 60 cm sided hexagon was marked on the floor with tape, with six rectangular signals (one for each side, consecutively numbered from S1 to S6) positioned 18 cm outside and away from the centre of each side. A force platform (Kitsler 9286BA, Kistler Group, Winterthur, Switzerland) was positioned at the centre of the hexagon to calculate the time employed to finish the task. The starting position was at the centre of the hexagon facing signal S6 with their knees bent approximately 45° [10]. The participant started the test immediately after a verbal “GO” command. The participant hopped over the line toward signal S1 and then back to the inside of the hexagon, then over the line toward signal S2, and so on as quickly as possible. The participant was instructed to keep their feet together and land on the targets with their forefeet. One complete circuit occurred when the participant hopped over the line toward signal S6 and back to the centre. One trial consisted of three circuits. Total time was calculated from the force platform signal. Start event was detected when the vertical force component decreased by 5% of the participant weight and finished with the last contact with the force platform.

#### 2.2.6. Fatigue Protocol

In order to induce a fatigued state in the participants, the Functional Agility Short-Term Fatigue Protocol (FASTFP) was carried out. The FASTFP was selected because it uses common athletic skills for assessing lower extremity biomechanical changes throughout the fatiguing process from a more ecological perspective [18]. A quick functional fatigue protocol altered lower extremity mechanics [18]. The FASTFP included three CMJ at 90% of maximal vertical jump, step-ups on a 30 cm box during 20 s at a pace of 200 bpm, 3 squats to 90° of knee flexion, and a pro-agility shuttle run (5-10-5 agility run) [18,19]. This was repeated until the maximal fatigue was reached, which was considered when (1) the participant did not attain 90% of the maximal jump on all three vertical jumps for two consecutive fatigue sets or (2) achieved a heart rate plateau over three consecutive fatigue sets within 90% of the estimated maximal heart rate [19]. Heart rate was continuously monitored through a pulsometer system (Polar^®^, model M400, Polar Electro, Inc., Lake Success, NY, USA).

### 2.3. Statistical Analysis

Statistical analysis was performed using SPSS 19.0 (IBM Armonk, New York, NY, USA). After checking the normality of the variables (a Shapiro-Wilk test), a descriptive analysis of the data was performed. Then a two-way repeated measures ANOVA with fatigue (rested vs. fatigued) and leg (right vs. left) as within-subject factors, extensibility group (NEG vs. HTG) as between-subject factors, and time in the agility test, reaches in mSEBT, and indexes in DPSI as the dependent variables was carried out. After that, a *t*-test for independent measures was used to identify the specific differences between extensibility groups (NEG vs. HTG). To identify meaningful changes, confidence intervals of the differences (CI 95%) and effect sizes (ES) were calculated. ES was assessed using Cohen’s *d* [36], and was interpreted as small (<0.50), moderate (0.50–0.79), or large (≥0.80). Significance was set at *p* < 0.05.

## 3. Results

**Figure 2:**
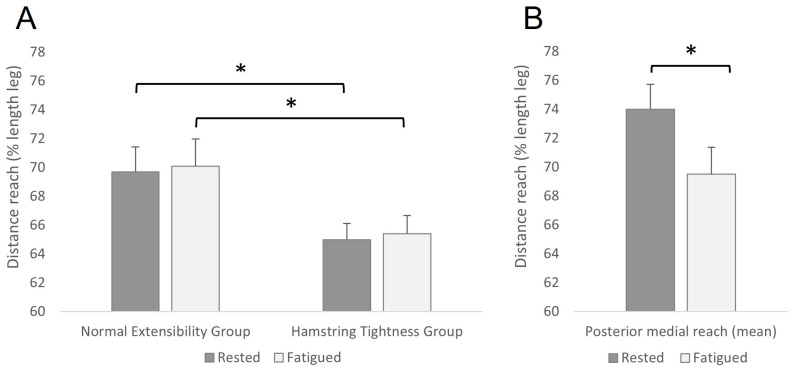
(**A**) Reach-differences between groups in the mean anterior direction. (**B**) Fatigue effects in posterior-medial direction in HTG. * Statistical differences (*p* < 0.05) in mSEBT = modified star excursion balance test.

Table 2 shows the descriptive values of the variables analysed in each of the tests. As no differences were found in any variable between legs for any study condition, all subsequent statistical analyses were conducted jointly and included both legs as one sample. The repeated measures ANOVA shows significant differences between the groups (NEG vs. HTG), as well as between the two fatigue states (rested vs. fatigued). Concretely, in the mSEBT without fatigue, HTG reached significantly less distance than NEG in the anterior direction both in the mean value (6.7% mean difference, *p* = 0.043, CI 95% = 0.56/8.81 cm, ES = 1.152) and in the maximum (7.0% mean difference, *p* = 0.001, CI 95% = 1.28/12.26 cm, ES = 1.333). Additionally, in the mSEBT in fatigued condition, HTG reached significantly less distance than NEG in the anterior direction in the mean value (7.3% mean difference, *p* = 0.004, CI 95% = 0.25/9.96 cm, ES = 1.046) and in the maximum one (7.6% mean difference, *p* = 0.002, CI 95% = 0.82/13.70 cm, ES = 0.871) (Figure 2A). A trend was observed on Ʃ Max (*p* = 0.053) during the rested condition, being lower the reach for the HTG compared to NEG. No significant differences were found in any other variable between HTG and NEG.

Regarding the fatigue effect, in the mSEBT the mean distance reached in the posterior-medial direction in HTG was significantly lower after the fatigue protocol (*p* = 0.035, CI 95% = 0.6/6.7, ES = 0.263) (Figure 2B). Similarly, in the DPSI test, the value of the medio-lateral stability index was higher (it means, lower stability) in NEG after the fatigue protocol (*p* = 0.021, CI 95% = −0.007/−0.001, ES = 0.526).

No differences were found in the hexagon agility test in any of the groups (NEG or HTG) and fatigue conditions (rested or fatigued) (*p* < 0.05). Only a trend was observed during the fatigued condition (*p* = 0.072) between HTG and NEG, being faster the HTG than NEG after the fatigue protocol.

## 4. Discussion

The aim of this study was to analyse the effects of hamstring tightness and fatigue on dynamic stability, agility, and reach directions. Our study shows a significant reduction in the anterior direction of the mSEBT in participants with hamstring tightness. In a study by Overmoyer and Reiser [36], similar ROM values were found and a significant reduction in the anteromedial direction of the mSEBT was reported. In our study, the slight changes in this direction were not significant. Overmoyer and Reiser [37] explained that the lack of dorsiflexion was the factor that most affects the anterior reach because when a participant wants to reach anteriorly with one leg, the stabilizing leg’s gastrocnemius and soleus restrict movement if they are tight. Although we did not measure ankle ROM, a study from Harty et al. [38] found a correlation between tight hamstrings and diminished ankle ROM. This is because tight hamstrings produce a premature muscular contraction of the posterior leg musculature, thus reducing ankle dorsiflexion. This phenomenon might explain why our participants with tight hamstrings had lesser values in the anterior direction of the mSEBT, both pre- and post-fatigue. According to Hubbard et al. [39], another factor that contributes to the lesser values in the anterior reach is the lack of hip flexion due to that, as we already mentioned, tight hamstrings affect the hip flexion values. Another possible explanation for the differences found in the study could be related to hip flexion during the execution of the mSEBT, where it has been shown that the position of the hip in the anterior position in the mSEBT is more flexed at the maximum point of reach (27.94° ± 13.84°) compared to the original version of the SEBT (20.37° ± 18.64°) [40], so participants with muscle restriction due to hamstring shortening will have reduced hip flexion and, consequently, the anterior reach will be reduced too.

Despite inducing fatigue, there was no significant change in stability. It could be related to a possible compensatory response to use the larger hip muscle extensors, suggesting that the lower extremity is able to adapt to fatigue though altering kinematics at impact during a single land task and redistributing work to larger proximal muscles, similar to what happens with landings from different heights after a local fatigue protocol [41]. A previous study with first division university football players showed that, after performing the FASTFP (fatigue protocol), kinematic changes are observed at the contact instant increasing knee internal rotation and reducing knee and hip flexion [19]. These adaptations could justify the absence of changes found in the dynamic levels of stability.

Another significant finding was that all participants, extensibility aside, presented longer times to stabilize the body in the mediolateral direction during the jump landing DPSI after the fatigue protocol. This result is similar to that found in the study by Pau et al. [42], in which they analysed the changes in COP on a static one-leg balance test in adolescent football players after an RSA fatigue protocol, finding longer stabilization times in all directions after fatigue. The reasons why we did not find any significant changes in other directions could be because the scoring values are not the same among the tests, the fatigue protocol was different, and our test required dynamic stabilization. Another factor that should be taken into consideration is the age of the participants. While Pau et al. [42] measured adolescent athletes, our participants were college students. It has been proven that the postural control system does not develop completely until after the teenage years [42], and our participants were well past those years, so this could be a probable reason why the global stability index was not significantly affected.

Increases in displacement in the mediolateral direction are due to peroneal muscle latency [43]. The increased time reaction of the peroneal is associated with delayed muscular force production which causes an increment in the time needed to stabilize in the mediolateral direction and, therefore, puts the ankle joint at risk of injury since ankle sprains occur mainly in the frontal plane [44]. This test has been able to predict who is prone to ankle injuries in elite adolescent athletes [45]. Instability in the frontal plane is not only a risk factor for ankle injuries, but it also supposes a risk for the knee joint. Increments in the valgus and varus angle of the knee occur at the frontal plane, and excessive angles are a risk factor for medial and lateral collateral ligaments and for anterior cruciate ligament injuries [46].

Our results could be explained by many factors. Firstly, anticipated postural adjustments (APAs) are functional adaptations of the motor system to maintain postural stability in the presence of fatigue [47]. It has been shown that postural muscles, especially the paraspinal muscles, are more frequently involved in APAs than leg muscles. So, as the fatigue protocol used in our study is focused mainly on the legs, maybe the paraspinal muscles were not fatigued enough to find changes in the rest of the dynamic postural stability variables. Secondly, another factor to take into account is that our participants maintained their eyes open because our test required jumping over an obstacle. It has been proved that visual input effectively compensates for the motor system deficiency to control balance [48]. Since the postural system is composed of different receptors, it is accepted that by affecting one system, another one will compensate to avoid a postural control collapse. Even with the eyes opened, some studies have analysed the visual system contribution to proprioception, and it is estimated that, in the presence of muscular fatigue, the visual system can compensate when the objective trying to be reached is at a distance of no more than four meters [49]. Since in the DPSI performed in our study participants should jump on the centre of the force platform from a 70 cm distance, the visual system could be able to compensate postural control deficits caused by fatigue.

Paillard [47] states that postural disturbances depend on physiological disturbances (e.g., central fatigue), mechanical impacts, and the level of dehydration (based on the possibility or not of rehydration). Dickin and Doan [50] found that a 30% loss of maximal voluntary muscular contraction of the lower extremity, measured by an isokinetic test, affected postural control. In our study, we measured a loss of maximal power of more than 10%, so possibly our protocol was not long enough to provoke significant postural deficiencies. It is also true that the postural control system returns to baseline faster than muscular force [42]. This is another important factor to consider in our protocol because, even though the participants performed the first attempt of DPSI immediately after the fatigue protocol, they had at least 10 s of rest between jumps while the force platform recalibrated. Moreover, we need to take into account the jumps that had to be discarded. By the time the participants reached the fifth and sixth jumps their heart rate and respiration rates were lower than 90% of their maximal capacity. Therefore, maybe the fatigue protocol used in our study was not long enough to provoke central fatigue and to reduce postural system compensations.

The results of the hexagon agility test did not show significant differences, neither between groups nor comparing pre- and post-fatigue. These results are not similar to the ones observed by García-Pinillos et al. [6] where participants with greater flexibility demonstrated more power, speed and agility than participants of the same age and sport but with lesser hamstring flexibility. The author explained that this is because a muscle with greater extensibility demonstrates faster rates of shortening and stiffness in the muscular tendon unit. From the authors point of view, there are previous studies that have shown that the reduction in joint ROM associated with the shortening of the hamstring musculature does not entail a reduction in muscle stiffness [51], probably because the shortened hamstring musculature does not reflect that it is due to an increase in stiffness [22]. In the hexagon agility test, as stiffness was not compromised and central fatigue was not reached, we believe that these are the reasons why there are no differences in sports performance.

The study is not without limitations. Although our sample size was similar to other studies that analysed dynamic stability, agility or fatigue effects [18,31,33,50], perhaps a higher sample size could explain more precisely the relationship among hamstring tightness, fatigue, dynamic stability and agility. Furthermore, as has been already explained, maybe the fatigue protocol was not long enough to cause central fatigue and therefore affect dynamic stability. Additionally, even though fatigue protocol has a specific criterion to determine when maximal fatigue state has been reached, other direct techniques, e.g., lactate analysis, could be more accurate to evaluate the fatigue level. Finally, although the tests employed to evaluate passive and active hamstring extensibility are reliable and two examiners are required to carry it out, these assessments could be influenced by participants’ subjectivity and stretch tolerance. Therefore, we think it would be interesting to carry out more research in the future with a higher number of participants and using direct methods to register fatigue levels.

## 5. Conclusions

Our results suggest that hamstring extensibility affects dynamic balance in the mSEBT. This test has been shown to be a predictor of future injuries and should be taken into account when designing personalised conditioning programs, especially in sports that require static balance on one leg. For more dynamic sports, when fatigue is not already present, extensibility does not seem to affect the ability to stabilize the body. However, in a fatigued state, independently from the extensibility level, it seems to take more time to stabilize the body in a mediolateral direction. Therefore, the conditioning program should be meticulously designed so the athlete can resist fatigue. We believe our fatigue protocol was not long enough to simulate a real team sports competition, and that is why our participants did not show a significant decrement in stability scores.

## Figures and Tables

**Table 1:** Characteristics of the participants.

	Normal Extensibility Group (*n* = 9)		Hamstring Tightness Group (*n* = 10)	
	Mean	SD	Mean	SD
Age (y)	23.0	4.3	21.4	2.2
Weight (kg)	75.3	10.7	74.5	7.4
Height (cm)	179.3	9.5	178.3	8.5
PSLR (°)	82.2 *	12.4	64.0 *	4.9
AKE (°)	150.6 *	10.0	135.1 *	5.5

PSLR = Passive Straight Leg Raise test; AKE = Active Knee Extension test. * Significant differences (*p* < 0.05) between normal extensibility group and hamstring tightness group.

**Table 2:** Descriptive results of group (NEG vs. HTG) and fatigue (rested vs. fatigued) effects on agility and dynamic stability variables.

	Normal Extensibility Group				Hamstring Tightness Group			
	Rested		Fatigued		Rested		Fatigued	
	Mean	SD	Mean	SD	Mean	SD	Mean	SD
Hexagon Agility test (s)	13.0	1.5	13.2	1.2	12.3	1.6	12.0	1.2
mSEBT test								
X_Ant (cm)	69.7 †	5.2	70.1 †	5.6	65.0 †	3.5	65.4 †	4.0
X_PL (cm)	71.8	2.4	70.3	2.8	71.7	6.5	72.5	6.0
X_PM (cm)	73.8	4.1	72.8	2.9	74.0 #	4.4	69.5 #	8.6
M_Ant (cm)	95.9 †	6.8	93.1 †	7.7	88.6 †	4.9	87.6 †	5.8
M_PL (cm)	98.0	3.2	96.3	3.7	98.0	11.5	96.0	10.6
M_PM (cm)	101.0	5.4	99.7	4.0	99.0	13.0	97.3	10.9
Ʃ Max (cm)	298.5	11.5	290.8	11.2	287.6	26.5	285.6	24.7
DPSI *								
VSI	1.0368	0.0050	1.0367	0.0053	1.0367	0.0059	1.0372	0.0043
MLSI	0.0257 #	0.0063	0.0308 #	0.0080	0.0260	0.0041	0.0273	0.0043
APSI	1.0957	0.0216	1.0933	0.0195	1.0962	0.0207	1.0878	0.0203
DPSI	1.5063	0.0120	1.5033	0.0100	1.5042	0.0238	1.5034	0.0156

* dimensionless; X = mean; M = maximum; Ant = anterior; PL = posterior-lateral; PM = posterior-medial; VSI = vertical stability index; MLSI = medio-lateral stability index; APSI = anterior-posterior stability index; DPSI = dynamic postural stability index. † Significant differences (*p* < 0.05) between normal extensibility group and hamstring tightness group. # Significant differences (*p* < 0.05) between rested and fatigued conditions.

## Data Availability

Not applicable.
